# Redo TAVR With Fluoroscopic-Free Surgical Resection of Prosthetic Valve Leaflets Under Direct Vision (SURPLUS) Technique

**DOI:** 10.1016/j.jacasi.2026.07.008

**Published:** 2026-08-20

**Authors:** Louise Bik-Yu Ting, Cheuk-Pang Wong, Chun-Ka Wong, Simon Cheung-Chi Lam, Inderjeet Bhatia, Daniel Tai-Leung Chan

**Affiliations:** a Department of Cardiothoracic Surgery, Queen Mary Hospital, Hong Kong SAR, China; b Cardiology Division, Department of Medicine, School of Clinical Medicine, Li Ka Shing Faculty of Medicine, The University of Hong Kong, Hong Kong SAR, China; c Cardiology Division, Department of Medicine, Queen Mary Hospital, Hong Kong SAR, China; d Department of Surgery, School of Clinical Medicine, Li Ka Shing Faculty of Medicine, The University of Hong Kong, Hong Kong SAR, China

**Keywords:** leaflet modification, redo transcatheter aortic valve replacement, SURPLUS, transcatheter aortic valve replacement, valve-in-valve transcatheter aortic valve replacement

## Abstract

We report a fluoroscopic-free approach to the Surgical Resection of Prosthetic Valve Leaflets Under Direct Vision (SURPLUS) technique. With expanding transcatheter aortic valve replacement (TAVR) volumes and broader indications, a failed transcatheter prosthesis is typically managed by redo TAVR. However, when bilateral coronary obstruction risk is prohibitively high and transcatheter leaflet modification techniques such as Bioprosthetic Aortic Scallop Intentional Laceration to prevent Iatrogenic Coronary Artery obstruction or Undermining Iatrogenic Coronary Obstruction With Radiofrequency Needle, or coronary stenting, are not feasible, transcatheter aortic valve explantation remains an alternative, yet it is technically demanding and carries significant risk according to real-world data. SURPLUS was previously described as a hybrid option wherein the failed leaflets are excised while the original stent is left in situ, followed by surgical valve-in-valve deployment. Here, we describe a completely fluoroscopic-free execution of SURPLUS in an older patient with high bilateral coronary obstruction risk, detailing the method to determine implantation depth and achieve commissural alignment without fluoroscopic imaging. The procedure resulted in no coronary obstruction and demonstrated good immediate and 1-year outcomes.

## Graphical Abstract

**Figure:**
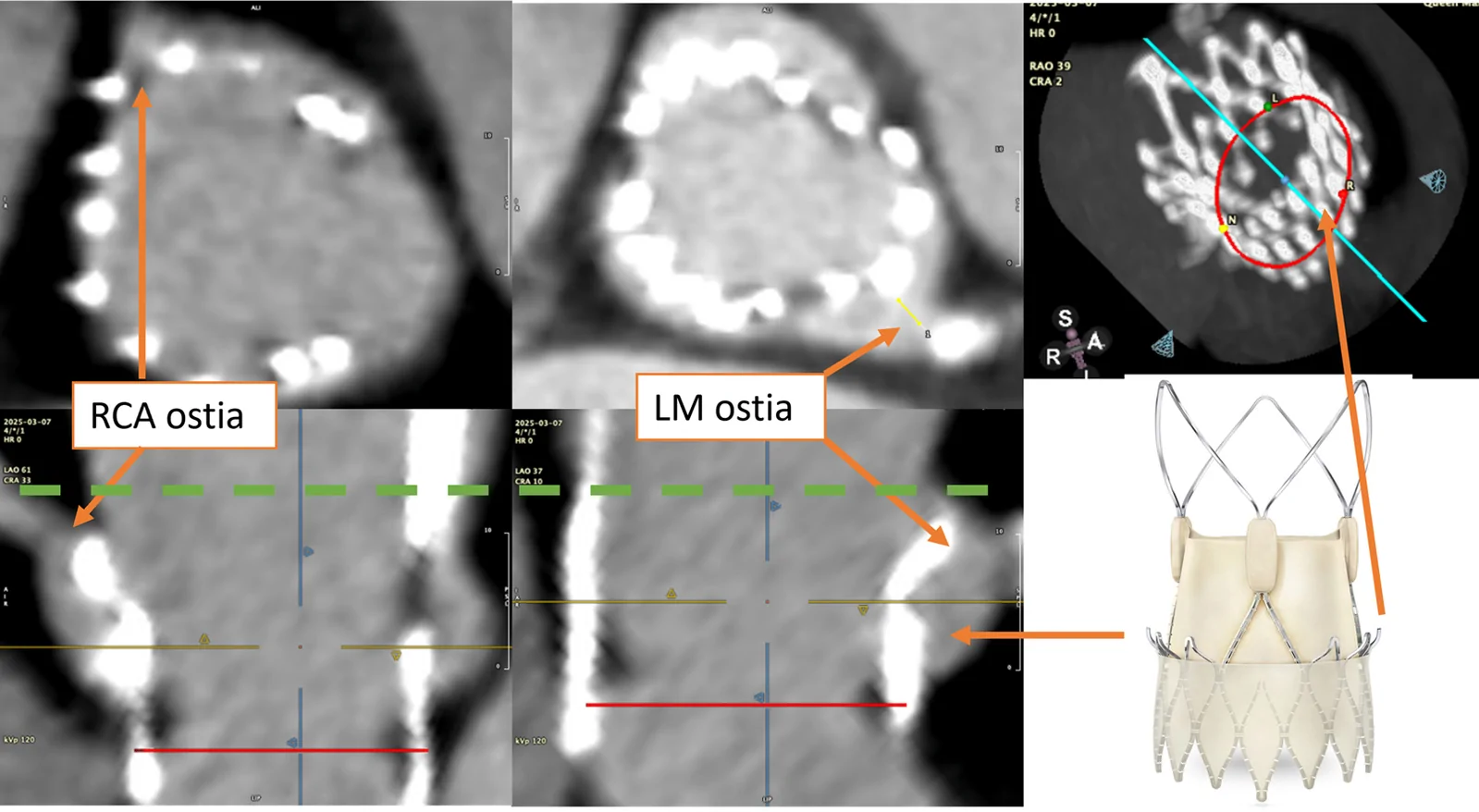

Transcatheter aortic valve replacement (TAVR) has been increasingly performed in the past 2 decades to treat both aortic stenosis and regurgitation. When these bioprostheses degenerate and develop severe valvular dysfunction, a redo TAVR procedure is usually performed.^1^ In patients with a high risk of coronary obstruction that is not amenable to leaflet modification or other transcatheter techniques,^2^, ^3^, ^4^, ^5^ surgical transcatheter aortic valve (TAV) explantation may be required. However, the TAV explantation procedure can be technically challenging because of adhesion to adjacent structures.^6^^,^^7^ The Surgical Resection of Prosthetic Valve Leaflets Under Direct Vision (SURPLUS) technique was developed to serve as an alternative surgical technique to treat patients with failed TAV and high coronary obstruction risk.^8^ In this report, we describe a fluoroscopic-free approach for performing the SURPLUS.

## Case presentation

An 80-year-old man had a history of hypertension, hyperlipidemia, bronchiectasis, atrial fibrillation, and coronary artery disease with percutaneous coronary intervention performed. He had previously undergone TAVR with size S ACURATE Neo (Boston Scientific) for severe aortic stenosis. He remained well until he developed progressive shortness of breath reaching NYHA functional class III, 7 years later. His N-terminal pro B-type natriuretic peptide level was elevated at 1,529 pg/mL. Echocardiography showed severe transvalvular aortic regurgitation, severe mitral regurgitation (effective regurgitant orifice area of 41 mm^2^), moderate tricuspid regurgitation (vena contracta width of 6 mm), and normal biventricular function. Computed tomography (CT) coronary angiogram revealed only minor coronary artery disease. Pre-TAVR electrocardiography-gated CT revealed an annulus area of 332 mm^2^ and an area-derived diameter of 20.6 mm. Both coronary ostia were determined to be at high risk for obstruction. Valve-to-coronary distances were 3 mm and 2 mm, respectively. Valve-to-sinotubular junction distance was nearly 0 mm. Coronary heights were only 4 mm and 2 mm, respectively. As the locator of the ACURATE Neo was anatomically lower than the coronary risk plane, it was decided that during direct vision deployment, the operative surgeon would aim to position the upper edge of the SAPIEN 3 Ultra Resilia (S3UR) (Edwards Lifesciences) slightly above the index valve locator (Figure 1).

**Figure 1:**
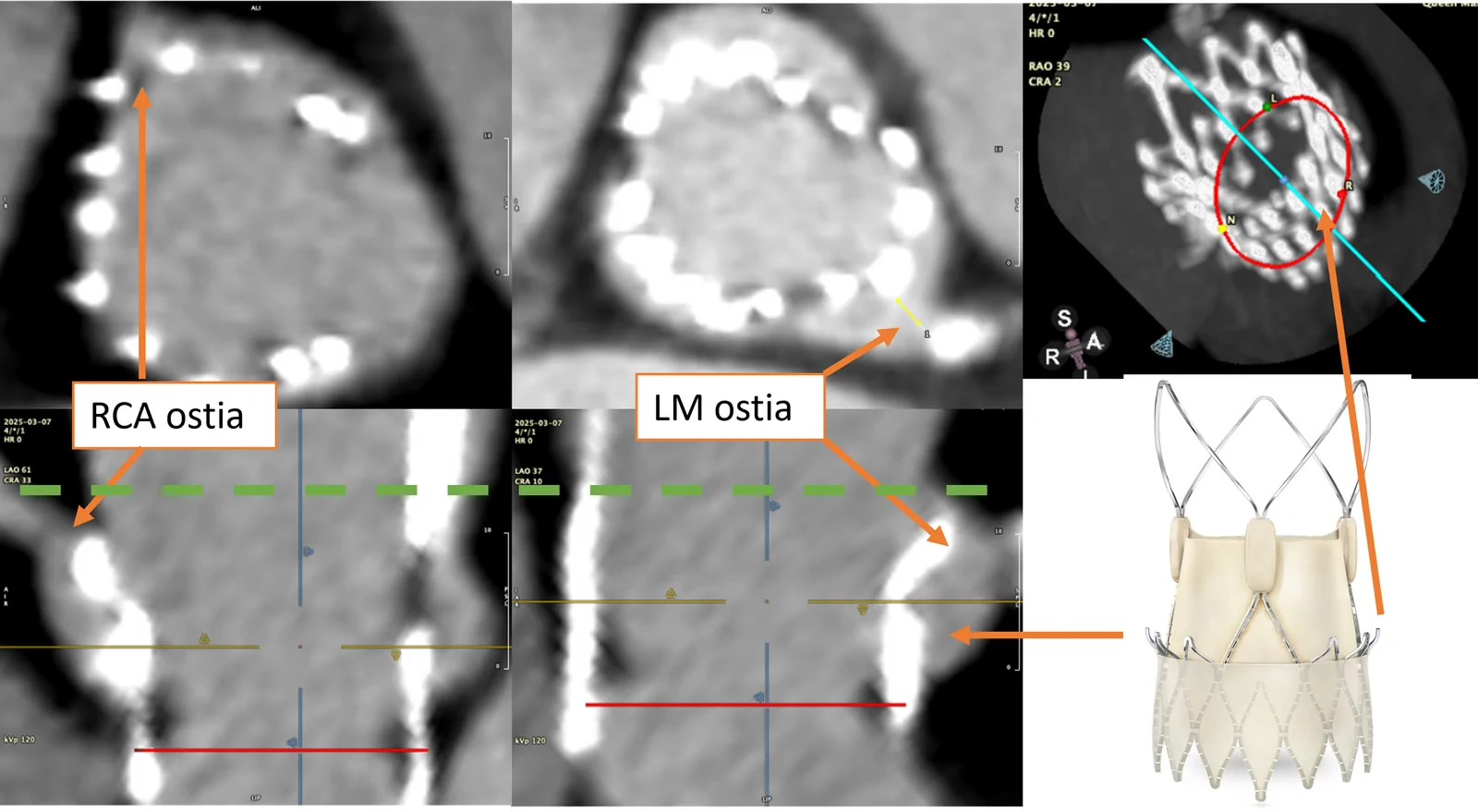
Preoperative Computed Tomography The valve-to-coronary distances of left and right coronary ostia were 3 mm and 2 mm, respectively. The neo-skirt plane (green dotted line) was projected to be higher than the coronary risk plane. The locator of ACURATE Neo, which is lower than the coronary plane, acted as a landmark for the second transcatheter aortic valve deployment during open surgery. LM = left main; RCA = right coronary artery.

The patient's clinical status and anatomy were reviewed by the heart team. It was determined that bilateral leaflet modification or other transcatheter coronary protection techniques would not be sufficient to prevent coronary obstruction in a redo TAVR procedure. The final consensus was to perform the SURPLUS procedure to treat the failed TAV. A watchful waiting strategy would be adopted for the mitral and tricuspid valve diseases.

## Fluoroscopic-free SURPLUS technique

A median sternotomy was made, with cardiopulmonary bypass initiated using arterial cannulation at the distal ascending aorta and a 2-stage venous cannula at the right atrium. The heart was arrested uneventfully using direct coronary ostial cardioplegia after opening the ascending aorta above the stent of the previous ACURATE Neo valve. The leaflet of the ACURATE Neo was found to be torn at the commissure between the right and left cusps (Figure 2). The leaflets of the previous TAV were then removed, while preserving the stent. A size 26-mm S3UR valve was prepared and deployed under direct vision, ensuring that the upper end of the S3UR stent remained slightly above the locator of the ACURATE Neo during slow balloon inflation. The valve was checked for proper commissural alignment and positioning. The aorta was then closed in standard surgical fashion. A size 35 mm AtriClip (AtriCure) was used to close the left atrial appendage. The wound was closed after meticulous hemostasis. The total duration of the surgery was 95 minutes, with a cardiopulmonary bypass time of 35 minutes and an aortic cross-clamp time of 24 minutes.

**Figure 2:**
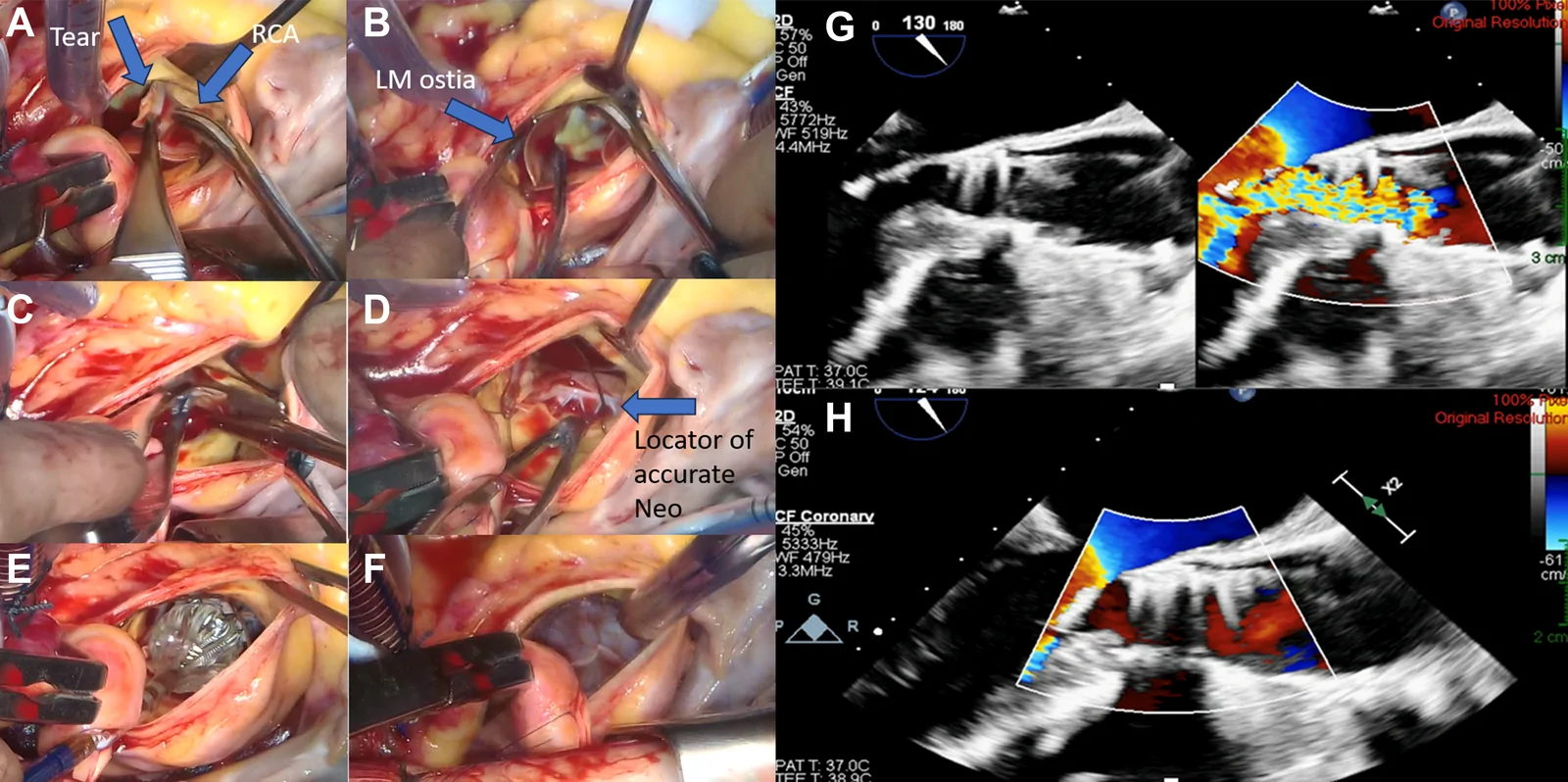
Fluoroscopic-free SURPLUS Technique (A) Degenerated index valve with tear at left-right commissure. Tip of scissor positioned at the right coronary ostium, in proximity to the leaflet. (B) Index valve leaflets covering left and right ostia during opening, demonstrating high coronary risk. (C) Leaflet excised while leaving behind the stent. (D) Locator of index valve, which is lower than the coronary ostia, acted as a landmark during deployment. (E) Commissural alignment checked before balloon expansion. Upper end of the second valve maintained to be slightly above the locator. (F) Second valve sat well with the commissure aligned. (G-H) Pre- and post-echocardiography showing resolution of regurgitation. Abbreviations as in Figure 1.

Intraoperative transesophageal echocardiography showed that the new TAV had no paravalvular leak and the mean gradient was 8 mm Hg (Figure 2). The postoperative course was uneventful. The patient stayed in the intensive care unit for 1 day and was discharged 9 days after the surgery. An echocardiogram performed 3 weeks after surgery showed a functioning TAV with no regurgitation, and the mean gradient was 6 mm Hg. Both mitral (effective regurgitant orifice area decreased to 30 mm^2^) and tricuspid regurgitation (vena contracta width decreased to 4 mm) showed improvement. At 1-year follow-up, the patient was in NYHA functional class I and echocardiography demonstrated functionally stable TAV.

## Discussion

With expanding indications and guidelines supporting the use of TAVR, the number of TAVR procedures has surpassed the number of surgically implanted valves in many countries.^9^ Although TAVR is increasingly performed in patients aged 65 years or younger, questions remain regarding valve durability. While long-term data are still lacking, echocardiographic follow-up of TAV beyond 5 years has shown a 3.3% valve failure rate, comparable to surgical bioprostheses.^10^ TAV explantation and redo TAVR have emerged as some of the fastest-growing procedures in recent years.^11^ Both procedures pose greater technical difficulty and higher risk than the initial intervention. A 2021 report from the EXPLANT-TAVR Registry demonstrated a high 30-day mortality of 13.1%.^6^

Besides nonstructural valve deterioration (eg, infective endocarditis, paravalvular leak, patient prosthesis mismatch), the risk of coronary obstruction is the most common reason to exclude patients from a redo TAVR. Sinus sequestration due to previous leaflets, supra-annular or long valve design, and commissural malalignment are common underlying reasons.^1^ There are several transcatheter strategies to mitigate the risk of coronary obstruction. Bioprosthetic Aortic Scallop Intentional Laceration to prevent Iatrogenic Coronary Artery obstruction (BASILICA) is a technique that lacerates the culprit leaflet using an electrified wire to achieve leaflet splay before second TAVR valve implantation.^2^^,^^3^ Undermining Iatrogenic Coronary Obstruction With Radiofrequency Needle (UNICORN) is an alternative technique that involves traversal of the target leaflet using electrified wire, followed by dilation of the de novo created fenestration using coronary and peripheral balloon. Thereafter, a balloon-expandable valve is inserted into the fenestration for intraleaflet deployment. This would split and spread the leaflet away from the coronary ostia.^4^^,^^5^ Although bilateral leaflet modification, such as bilateral BASILICA (“doppio BASILICA”) and bilateral BASILICA-UNICORN, is technically challenging, it is still feasible if experienced operators are available.^5^ Nevertheless, this patient was anatomically unfavorable for bilateral leaflet modification. Bilateral leaflet modification involving BASILICA in at least one side was not considered in this patient because the leaflet splay in redo TAVR scenario with extremely low valve-to-coronary distance might be insufficient to mitigate coronary obstruction risk. On the other hand, UNICORN with intraleaflet balloon-expandable valve deployment could only be performed unilaterally. Although other coronary protection methods such as chimney or snorkel stenting have effectively protected coronary blood flow in other reported cases,^12^^,^^13^ they were anticipated to be insufficient in this patient because of the extreme risk of coronary obstruction.

In situations in which redo TAVR is not possible, TAV explantation serves as the last resort. It is currently one of the fastest-growing cardiac surgical procedures in the United States; however, it carries a high operative and perioperative mortality compared with redo TAVR.^14^ Because of the tall design of TAV, explantation could result in damaging surrounding structures, resulting in a high rate of concomitant procedures.^14^ Patients requiring explantation are often old, frail, and in a more critical condition due to the underlying indications such as endocarditis.^15^ Another approach to address this technical challenge is the SURPLUS procedure first reported by Pirelli and colleagues in 2021.^8^ The SURPLUS technique can be considered a hybrid procedure that eliminates the complication of coronary obstruction resulting from previous valve leaflets, while avoiding the complications associated with valve explantation, including damage to the aortic root, ascending aorta, interventricular septum, and aortomitral curtain; and higher risk of heart block and other complications related to a long cardiopulmonary bypass time. Our heart team has performed more than 50 cases with leaflet modification, including 10 with bileaflet involvement and 8 TAV explantations. However, this is the first patient for whom we believe the SURPLUS technique might be the safest approach. Ultimately, the cross-clamp and bypass times (24 minutes and 35 minutes, respectively) in this case were much shorter even when compared with a regular first-time surgical aortic valve replacement. Combined with a favorable clinical outcome, this approach represents a feasible alternative to the complicated redo TAVR.

Although the previous report involved the use of intraoperative fluoroscopy, which might pose logistical and technical difficulties, this case demonstrated the ease of redo TAVR deployment by direct vision. The success of our approach relies on identifying important landmarks from the preoperative CT, such as previous TAVR stent structure, distance from the distal stent, or the coronary ostia themselves. The choice of a balloon-expandable valve in this case also simplifies the deployment process as it can be mounted on the balloon and thereby bypass the alignment process. Moreover, because the valve expands by bottoms-up foreshortening, the surgeon only needs to maintain a stable position of the upper stent during inflation. Commissural alignment also can be easily achieved before the start of inflation. Despite its advantages, SURPLUS is not suitable for patients with endocarditis or patient-prosthesis mismatch.

## Conclusions

In conclusion, SURPLUS is a safe and novel technique for patients requiring reintervention after TAVR who are at high risk of coronary obstruction, while avoiding the risk of aortic wall damage and allowing shorter bypass time compared with explantation. Although it is more invasive than transcatheter approaches, it is a safe and effective alternative in selected patients.

### Ethics Statement

Ethical approval was obtained from the Institutional Review Boards of the University of Hong Kong and the Hospital Authority Hong Kong West Cluster (HKU/HA HKW IRB; UW 12-177).

## Funding Support and Author Disclosures

The authors have reported that they have no relationships relevant to the contents of this paper to disclose.
